# From trickle to flood: the large-scale, cryptic invasion of California by tropical fruit flies

**DOI:** 10.1098/rspb.2013.1466

**Published:** 2013-10-07

**Authors:** Nikos T. Papadopoulos, Richard E. Plant, James R. Carey

**Affiliations:** 1 Laboratory of Entomology and Agricultural Zoology, School of Agricultural Sciences, University of Thessaly, Phytokoy Street 38446 N. Ionia (Volos), Magnisias, Greece; 2 Department of Plant Sciences and Biological and Agricultural Engineering, University of California, Davis, CA 95616, USA; 3 Department of Entomology, University of California, Davis, CA 95616, USA; 4 Center for the Economics and Demography of Aging, University of California, Berkeley, CA 94720, USA

**Keywords:** Tephritidae, invasion biology, subdetectable populations, eradication

## Abstract

Since 1954, when the first tropical tephritid fruit fly was detected in California, a total of 17 species in four genera and 11 386 individuals (adults/larvae) have been detected in the state at more than 3348 locations in 330 cities. We conclude from spatial mapping analyses of historical capture patterns and modelling that, despite the 250+ emergency eradication projects that have been directed against these pests by state and federal agencies, a minimum of five and as many as nine or more tephritid species are established and widespread, including the Mediterranean, Mexican and oriental fruit flies, and possibly the peach, guava and melon fruit flies. We outline and discuss the evidence for our conclusions, with particular attention to the incremental, chronic and insidious nature of the invasion, which involves ultra-small, barely detectable populations. We finish by considering the implications of our results for invasion biology and for science-based invasion policy.

## 1. Introduction

Tropical fruit flies (Tephritidae), such as the Mediterranean fruit fly (*Ceratitis capitata*) from Africa, the oriental fruit fly (*Bactrocera dorsalis*) from Asia and the Mexican fruit fly (*Anastrepha ludens*) from the Americas, are recognized by entomologists as among the most destructive agricultural insect pests in the world [1,2]. Because of tephritids' economic importance, US states such as California—considered by both the US Department of Agriculture (USDA) and the California Department of Food and Agriculture (CDFA) to be free of these pests, but with climates favourable to their establishment—invest heavily in measures to keep tephritids from becoming established. These steps include restricting importation of commodities that originate in regions with ongoing tephritid outbreaks, requiring post-harvest treatments for imported fruits and vegetables grown in areas where the pests are endemic or established, maintaining large-scale monitoring programmes for early detection, supporting preventive release programmes of sterile flies to pre-empt establishment, and launching eradication campaigns to eliminate pest populations once discovered. Indeed, 90% of the eradication projects (243 of 274) initiated in California between 1982 and 2007 were directed against tropical fruit flies (see electronic supplementary material, table S1).

The historical challenges posed by the fruit fly threat to California are similar to those posed by many other invasive insect species [3]. For example, the propagule pressure of fruit flies resulting from the ever-increasing movement of people and products [4–6] is an ongoing challenge posed by all invasive species. Similarly, global warming has resulted in the expansion of pest ranges worldwide [7]. Fewer frost days, longer growing seasons, more heat waves and greater frequency of warm nights in California [8,9], combined with an abundance of suitable hosts [2] in both urban and commercial environments, create ideal conditions for a wide range of species, particularly tropical tephritids, to successfully invade.

Two aspects of California's fruit fly invasions are unique, however. First, in most years and locations, fruit fly detections are extremely rare because of a combination of the slow population growth of newly introduced species and of population suppression from intervention programmes. This combination of elements makes it difficult to decipher patterns in detections, because there are few ‘dots’ to connect, and small numbers of captures separated in both time and space may give the illusion that previously detected populations have been eliminated. Second, an unprecedented number of pest tephritids have been detected in California in recent decades, including a more than eightfold increase in the number of species (i.e. *n* = 2 in 1954; *n* = 17 in 2012), and thousands more flies have been captured in California than in all other US mainland states combined. We are unaware of any other single taxonomic group (family) that consists of such a large number of economically important invasive species that are continually reappearing in the same region.

Our broad goal in this paper is to bring principles of invasion biology [3,10,11], mapping techniques and quantitative methods to bear on detection and interception data to answer questions about the residency status of tropical fruit flies captured in California. We show that, despite the due diligence, quick responses and massive expenditures of government agencies to prevent entry and establishment of these pests, virtually all of the species against which eradication projects were directed have been reappearing; several species reappear annually, and several others every 2–5 years. The preponderance of evidence supports the hypothesis that at least five and as many as nine species are established in the state. We discuss both scientific and practical implications of these findings.

## 2. Methods

### (a) Data sources

We obtained, from the California Department of Food and Agriculture (CDFA), historical detection data (1950–2011, in Excel spreadsheets) for all 17 tropical fruit fly species (see electronic supplementary material, figure S1 and table S1*a*,*b*) that have been trapped in California [12,13]. We also used the California Plant Pest and Disease Reports available online, medfly detection data in the database of J.R.C. (1975–1994) and the CDFA webpage for recent (2012) detections. Separate records were created for each tephritid adult, including its species, sex, date, mating status (females only) and precise location (latitude and longitude). We also examined information on larval finds obtained by ground crews searching for infested hosts in the 24–48 h window between capture of an adult fly and intervention. Although no tropical tephritid species was detected in the state until 1954, California fruit growers had been on high alert for tephritid introductions ever since the first medfly detection in Hawaii in 1910 [14,15].

### (b) Mapping

All detection data were entered into ArcGIS Map 10 (Esri, New York, NY) and transformed to WGS 84 coordinates. They were then re-projected to UTM coordinates of the appropriate zone. Finally, historical detections for each species were mapped at local, regional and state-wide scales.

### (c) Tephritid propagule pressure and climatically favourable regions

We used information on both domestic and international interceptions at ports of entry (see electronic supplementary material, tables S3–S6) to estimate relative propagule pressure in fruit-fly-friendly regions of California (all areas except far northern and alpine regions) relative to other regions of North America (southern states) and the Mediterranean Basin (all countries) that have climates favourable to tephritid establishment [12,16–20]. Information on tephritid interception pathways and field detections in fruit-fly-friendly regions other than California served as controls; that is, similar rates of tephritid interception at ports of entry for different regions do not ‘explain’ why there are ongoing field detections in one region (California) but not in the others. The relationship between port of entry and field detections must be considered to avoid misrepresenting correlation as causation (the *false-cause fallacy*), as has been carried out in the past (see citations in [21,22]).

### (d) Modelling

A natural estimate of the probability of capture in a region within *n* years of an initial detection is the fraction of detection years in that region in which the species is detected again within *n* years of the most recent detection. We computed this fraction for each species at two spatial scales: the county scale and a local scale based on the size of the exclusion area surrounding a detection. To define localities, we subdivided the area into a lattice of square cells of side length 14 km, a dimension that approximates nearly the same area (196 km^2^) as the federally mandated treatment area, a circle of radius 8 km (201 km^2^) around a fruit fly discovery. We considered each lattice cell to be a separate region. We analysed southern California and the Bay Area separately. There were sufficient data for a detailed analysis of two species, *B. dorsalis* and *C. capitata*, in the Bay Area, and of these two species plus *A. ludens* in southern California. Results of the analysis of these three species are reported in detail at all scales. Results for the other species are summarized graphically at the county level.

Although the recapture model was developed for forecasting recurrence, we also used it in a randomization trial to test a null hypothesis of random introduction against an alternative hypothesis of reoccurrence in a currently infested region. We separated the Bay Area and the Los Angeles area as above. For a given species in each area, we selected only lattice cells in which an infestation had been detected at least once. For example, for *B. dorsalis*, there are 30 and 77 such cells in the Bay Area and the Los Angeles region, respectively. We numbered from 1 to *n* the years in which a member of the species was captured, skipping years with no capture. For *B. dorsalis, n* = 27 and *n* = 45 for the Bay Area and Los Angeles region, respectively. Let *U* be a vector of length *n* − 1, and let *U_j_* = 1 if there is at least one cell in which a member of the species was captured in that cell in both years *j* and *j* + 1, and let *U_j_* = 0 if there is no such cell in year *j*. Let *N* equal the sum of the elements of *U*. Then the statistic *N* is a measure of the persistence of the infestation in the same cells. This model was used in a randomization test by comparing the actual observed value of *N* with values obtained under the null hypothesis of random introduction into the same set of cells each year. The analysis was carried out only for those species with sufficient data (at least 50 unique records) for a lattice-level recurrence analysis, as described in the previous paragraph.

## 3. Results

### (a) Historical overview of detections

**Figure 1.**
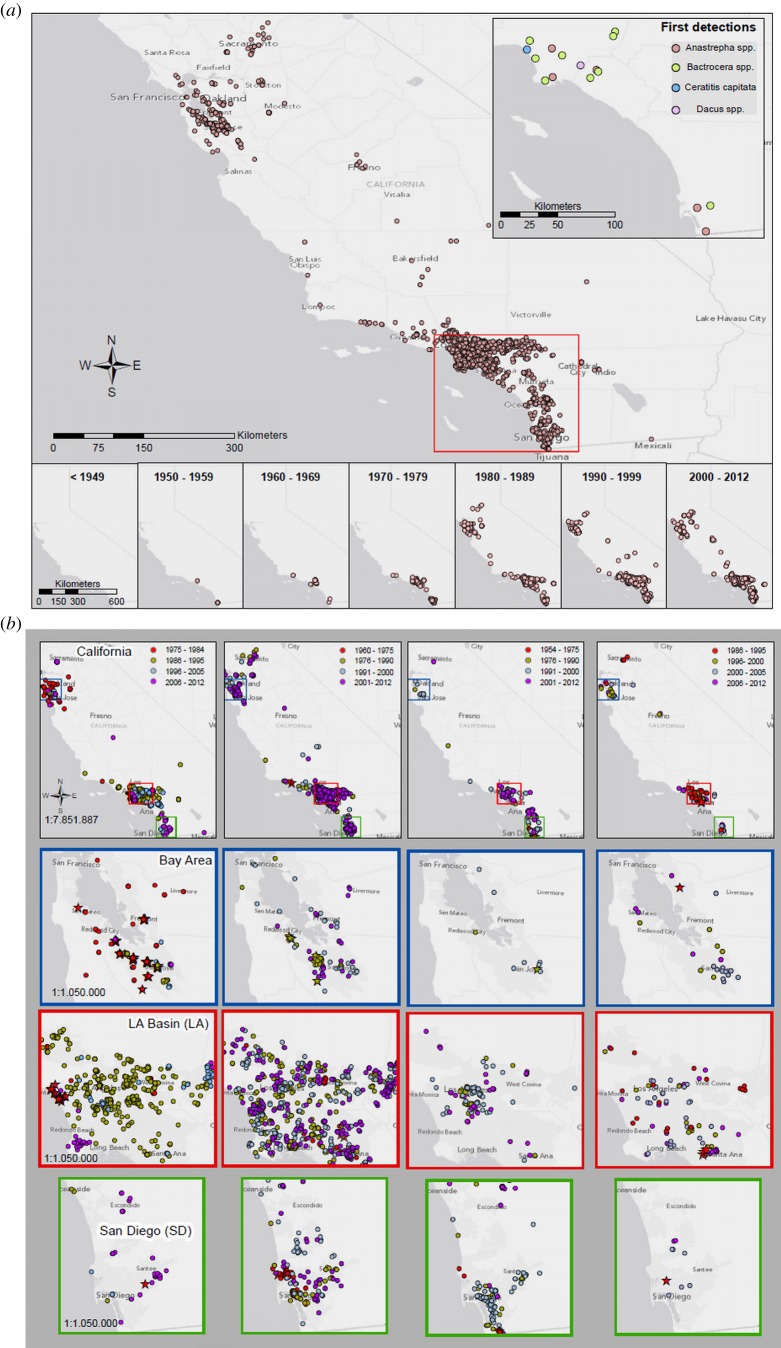
Locations of tropical fruit fly detections in California (i.e. each point represents one or more individuals detected at a single location and date). (*a*) Cumulative captures of all 17 tephritid species from the first detections in the 1950s to the most recent. Inset shows the locations of first detections for all species, and the mini-maps at the bottom depict the detections (non-cumulative) by decade. (*b*) Detection patterns of the four most frequently captured fruit fly species at the state level (top row) as well as in three regions: the San Francisco Bay Area, the Los Angeles Basin and the San Diego greater metropolitan area. Stars indicate location of initial regional captures. Maps show detection locations for (left to right) *C. capitata*, *B. dorsalis*, *A. ludens* and *B. correcta*.

Tephritids have been detected in nearly all regions of California where conditions are favourable for fruit fly establishment (table 1 and figure 1*a*; see also electronic supplementary material, figures S2–S18). Although the largest numbers of detections by far have been in the greater metropolitan areas of southern California, including the Los Angeles Basin and San Diego, a substantial number of flies were also detected in northern California, in the San Francisco Bay Area. Tephritids also began appearing in the state's main agricultural growing region, the Central Valley, which includes the Sacramento and San Joaquin Valleys, and the Imperial Valley. The non-random pattern of the invasions is reflected in the fact that 100% of first records for all species were in southern California (figure 1*a* inset), all but one of which were found in two regions: Los Angeles and San Diego. These regions contain only around one-third of the state's population, yet account for 100% of the tephritid first records.

**Table 1.** Tephritid fruit fly species in four genera (*Anastrepha, Bactrocera, Ceratitis* and *Dacus*) that have been detected in California to end of 2012, including scientific and common names, city, county, year of first detection, year last detected, and basic metrics of capture, including numbers of individuals, detections and cities infested. LA, Los Angeles; OR, Orange; SD, San Diego. Numbers of olive fly detections and cities are not included because CDFA decided that this species was too widespread and deeply entrenched to attempt eradication, and thus considered it established a few years after its discovery.

		city (county)	year				number		
species	common name	first record	first	last	span	detection years	adults (larvae)	detections	cities
1. *A. ludens*	Mexican fruit fly	San Ysidro (SD)	1954	2012	58	41	889 (295)	465	77
2. *B. cucurbitae*	melon fly	Westwood (LA)	1956	2011	55	15	34 (0)	28	17
3. *B. dorsalis*	oriental fruit fly	Anaheim (LA)	1960	2012	52	44	2266 (1755)	1558	244
4. *A. striata*	guava fruit fly (A)	San Ysidro (SD)	1963	2012	48	9	11 (0)	10	11
5. *A. obliqua*	West Indian fruit fly	Wilmington (LA)	1967	2005	42	6	7 (0)	7	8
6. *C. capitata*	Mediterranean fruit fly	Santa Monica (LA)	1975	2012	37	26	2068 (3884)	1417	169
7. *A. suspensa*	Caribbean fruit fly	San Diego (SD)	1983	2004	21	4	6 (0)	3	3
8. *B. zonata*	peach fruit fly	El Segundo (LA)	1984	2010	26	12	61 (0)	57	23
9. *B. tryoni*	Queensland fruit fly	La Mesa (SD)	1985	1991	6	2	2 (0)	1	2
10. *B. correcta*	guava fruit fly (B)	Westminster (OR)	1986	2012	26	23	131 (0)	126	59
11. *B. scutellata*	striped fruit fly	Los Angeles (LA)	1987	2010	33	6	15 (0)	15	8
12. *D. bivitattus*	African pumpkin fly	Cerritos (LA)	1987	1987	1	1	1 (0)	1	1
13. *A. serpentine*	Sapote fruit fly	Los Angeles (LA)	1989	1989	1	1	1 (0)	1	1
14. *B. latifrons*	Malaysian fruit fly	South Gate (LA)	1998	1998	1	1	1 (0)	1	1
15. *B. facialis*	no common name	La Verne (LA)	1998	1998	1	1	1 (0)	1	1
16. *B. oleae*	olive fly	Los Angeles (LA)	1998	2012	14	14	NA	NA	NA
17. *B. albistrigata*	white striped fruit fly	Pomona (LA)	2008	2009	2	2	9 (0)	9	4
							*Σ* 5503 (5934)	3700

California was free of any tropical fruit fly species before the mid-1950s (figure 1*a* mini-maps), despite the rapid growth of the fruit industry in the late nineteenth century and first half of the twentieth century, as well as relatively lax regulatory protocols at ports of entry [23,24]. Two species were detected during the 1950s (*A. ludens* in the greater San Diego area and *B. cucurbitae* in the Los Angeles Basin), followed by four more in the 1960s and 1970s. The tephritid situation in the state changed drastically in the 1980s because of: (i) continued reappearances and spread of previously detected species in metropolitan Los Angeles and San Diego; (ii) seven new species detected, raising the total in the state from six to 13 species (table 1); and (iii) first tephritid detections in northern California (figure 1*a*,*b*), including a massive, widespread medfly outbreak in the Bay Area [21,25,26].

Three new tephritid species captured in the 1990s raised the total in the state to 16. The economic stakes were elevated to a new level when one of these, the olive fly (*B. oleae*), was declared established, and several previously detected species appeared in the Central Valley growing region. Even though only one new species has been captured during the past 12 years, nine previously detected species have been recaptured repeatedly over ever-expanding areas (table 1 and figure 1*a*), including seven that have been captured multiple times during the past 3 years (excluding *B. oleae*). The magnitude and geographical scope of the recurrent detections are evident in maps in figure 1*b*, showing the historical records of the hundreds of state-wide, regional and local detections of the four most frequently captured species.

**Figure 2.**
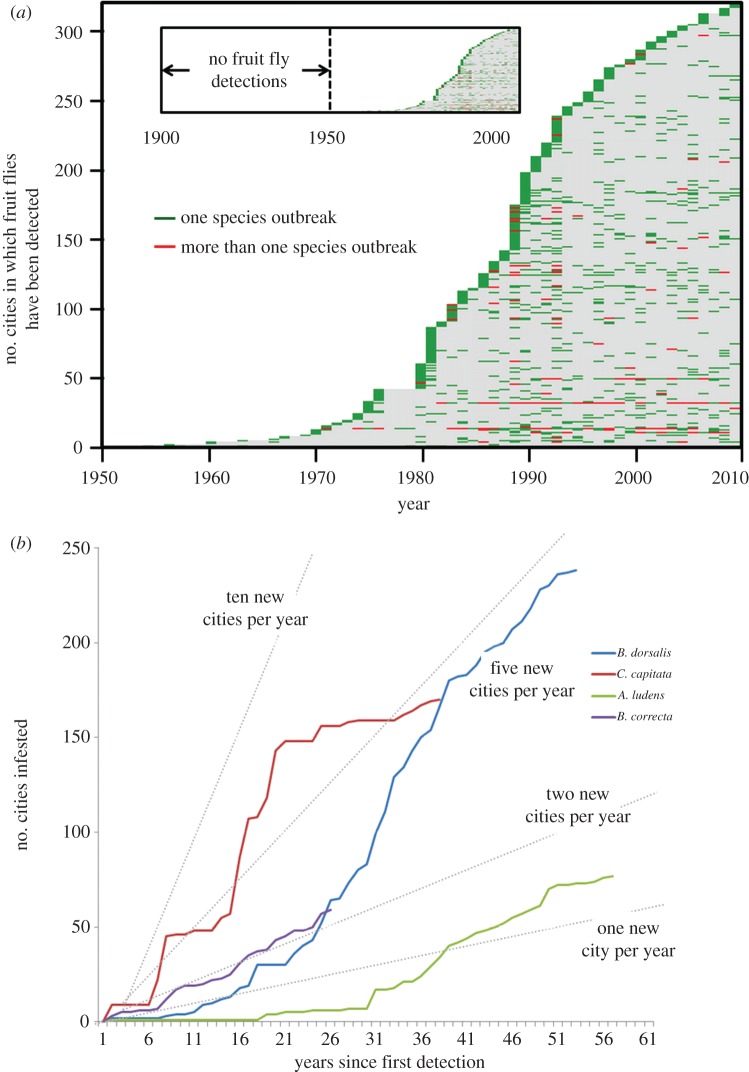
Appearance of tephritid fruit flies in Californian cities. (*a*) Event-history chart depicting the cumulative number of Californian cities that experienced one or more fruit fly infestations from 1950 to the present. Each horizontal line represents a Californian city, and coloured ticks depict the year in which one or more species was detected. Inset situates this schematic in a 100+ year context (California became a state in 1850). (*b*) Cumulative number of new Californian cities from which a detection of one of four different fruit fly species was reported. The abrupt levelling of the trajectory for *C. capitata* at 150 cities infested occurred at the same time that the California medfly preventive release programme was implemented in 1996.

Using the number of cities in which a tephritid species has been detected as a proxy for area infested, figure 2*a* shows that in 1960 there were only two California cities in which tephritids had been detected (one in the San Diego region and one in the Los Angeles region). However, by 1970 the number of cities with a tephritid detection had increased to 13, by 1990 to a remarkable 200 cities, and by 2010 to more than 300 cities. Although 10 different species (excluding the olive fly) contributed to these totals, *A. ludens*, *C. capitata* and *B. dorsalis* contributed the most, appearing in 77, 168 and 245 new cities, respectively, by 2012 (figure 2*b*; see also electronic supplementary material, video S1).

### (b) Reintroductions versus established populations

A long-standing explanation for recurring fruit fly detections is that flies are continually being reintroduced, either in cargo shipments or by people carrying infested fruit from fruit-fly-infested regions of the world [27–29]. We test this hypothesis and an alternative one to account for the recurring detections; both hypotheses were originally framed by Carey for the medfly [25,30] as (i) *reintroduction hypothesis*—recurring tephritid detections are due to repeated introductions—and (ii) *established population hypotheses*—recurring detections are due to resident fly populations. We assess the strength of these two hypotheses by comparing and identifying inconsistencies in relative numbers, diversity and frequency (or lack) of detections in California and in other fruit-fly-friendly regions.

#### (i) Absence of tephritid detections in most at-risk US states

Tephritids are intercepted at all airports across the USA (see electronic supplementary material, table S2) including all airports located in the southern states considered at risk for tephritid introductions. California ports of entry accounted for less than 20% of all insects intercepted in at-risk states (see electronic supplementary material, tables S3 and S4).

Assuming that insect interceptions (and specifically tephritid interceptions) can serve as proxies for the relative propagule pressure, if reintroductions were the primary source of detections, then the number of fruit fly detections in fruit-fly-friendly regions of the USA outside of California, compared with detections in California, should be roughly five to one, because California contributes about 20% of detections. Yet no tephritids were detected in the majority of states (i.e. all southern states) that are deemed at risk for fruit fly introduction [12,19,31] and maintain robust monitoring programmes (i.e. Arizona, Florida and Texas had relatively few detections).

#### (ii) High tephritid interception rate in European Union but near-absence of new species

Although the medfly and the olive fly are the only two tropical tephritid species that are long-term residents of fruitfly-friendly regions (southern countries) in the European Union (EU), interception rates of other tephritid species at ports of entry throughout the EU are quite high. One source of evidence for this is EUROPHYT, the European notification system for plant health interceptions. This system's database revealed that, of the total number of interceptions of harmful organisms in plants and plant products imported into the EU in 2011 (*n* = 1600), fully one-third (*n* = 534) were tephritids (see electronic supplementary material, table S6; see also [6]), and showed that, from 2007 to 2009, more than 700 individual tephritids in three genera (*Anastrepha, Bactrocera*, *Ceratitis*) and nine species not established in Europe were intercepted at Paris's International Airport (see electronic supplementary material, table S5). If the diversity and number of tephritid interceptions at the scores of international airports located in fruit-fly-friendly southern Europe, northern Africa and the Middle East are similar to those at the Paris airport, then the tephritid propagule pressure throughout this world region is far greater than in California. Yet, despite this pressure, with the exception of the peach fruit fly (*Bactrocera zonata*), discovered in 1998 in Egypt [32], no other tropical tephritids have been detected throughout the Mediterranean Basin for a century.

#### (iii) Evidence of breeding populations

Evidence of breeding populations in California is indicated by larval collections (table 1) for three species: (i) oriental fruit fly (*B. dorsalis*)—a total of 1755 larvae were collected in 169 locations over several periods totalling 21 years between 1974 and 2002; (ii) Mexican fruit fly (*A. ludens*)—a total of 295 larvae were collected at 15 locations in 4 years between 1995 and 2002; and (iii) medfly (*C. capitata*)—a total of 3884 larvae were collected in 572 locations over periods totalling 17 years between 1975 and 2009. Additional evidence for continuous populations of the medfly in California is in the papers by Meixner *et al.* [33] and Bonizzoni *et al*. [34], both of which showed that genetic analysis of captured flies over many years was consistent with continuous populations (see also electronic supplementary material, figure S21).

#### (iv) Repeat finds

**Figure 3.**
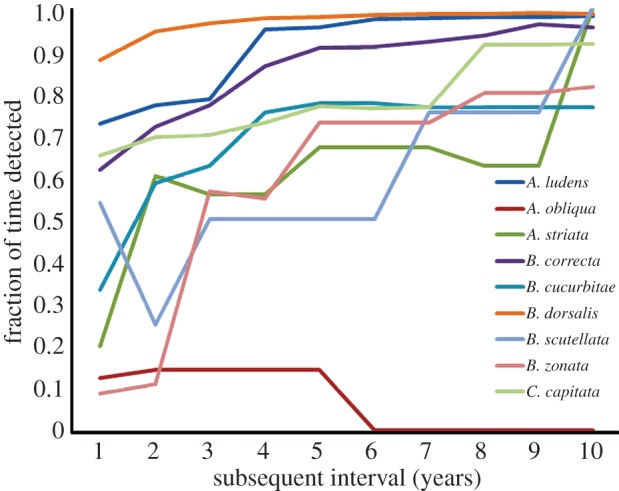
Estimated fruit fly recapture probabilities at the county level for nine fruit fly species in California in subsequent years (1–10 years) following a detection. For example, the probabilities of a repeat outbreak occurring for *A. striata, C. capitata* and *B. dorsalis* the first year following a detection are around 0.20, 0.65 and 0.88, respectively; the fifth year after a detection they are around 0.65, 0.75 and 0.98, respectively; and the 10th year after a detection they are around 1.00, 0.91 and 0.98, respectively.

At the *state level* from 1980 through 2012, one to two different tephritid species were captured every year (i.e. during 100% of this 33-year period), and the following numbers of species were captured during the percentages of this period indicated for each: three species, 97; four species, 78; five species, 72; six species, 62; and seven species, 25. In one of these years (1998), a remarkable 11 different species were captured, eight of which were repeats. At the *county level* (figure 3), depending upon species, the estimated probability of recapture after 1 year ranged from 0.1 to nearly 0.9, after 5 years from 0.5 to nearly 0.95, and after 10 years from 0.7 to near 1.0. At the *city level*, the frequency of repeat detections was high for many species, but extraordinarily so for three: 49 cities experienced repeat detections of *C. capitata* from two to 11 times, and 25 and 92 cities experienced respective repeat detections of *A. ludens* and *B. dorsalis* from two to 19 times. At the *lattice cell level* (14 × 14 km), the estimated probability of recapture in the Los Angeles area for *A. ludens* was 0.41 after 1 year, 0.70 after 5 years and 0.92 after 10 years. For *B. dorsalis*, these probabilities were 0.45, 0.79 and 0.94, respectively, and for *C. capitata*, they were 0.32, 0.55 and 0.64, respectively. In the Bay Area, the corresponding 1-, 5- and 10-year recapture probabilities were 0.12, 0.39 and 0.68, respectively, for *B. dorsalis* and 0.56, 0.59 and 0.88, respectively, for *C. capitata*.

An argument often used to account for the repeat finds in the same local area is that the same persons repeatedly return to California with infested fruit, reintroducing the species. However, the data do not support this argument, because there were no between-year detections on the same properties; in addition, this ‘reinfestation behaviour’ is not seen in other states and should not be unique to California returnees only.

#### (v) Randomization test of the null hypothesis of random introduction

In each case, the value of the observed recapture statistic *N* was compared with 999 values of this statistic computed under the assumption of random introduction in each year. In the Bay Area, the probability of obtaining an *N* statistic at least as large as the observed value was estimated as *p* = 0.038 for *B. dorsalis*. In the Los Angeles area, the probability was estimated as *p* < 0.001 for *B. dorsalis* and *A. ludens*, and *p* = 0.007 for *C. capitata*. These tests are anti-conservative (i.e. the *p*-values may be too low) because they ignore differences in both habitat suitability and trapping intensity among the lattice cells. Such differences would tend to increase the probability of capture in certain cells even in the case of random release into these cells, which would reduce the significance of the *N* statistic. Nevertheless, the results provide further support for the idea that the recapture pattern is not one that would be observed if the insects were being reintroduced each year.

## 4. Discussion

Several lines of evidence support the hypothesis that from five to nine tephritid species have become self-sustaining (and therefore established [35]) populations in the state (see electronic supplementary material, table S7 for list, establishment probability categories and summary of invasion metrics): their abrupt first appearance in the mid-1950s followed by high incidence of repeat detections, their marked seasonality (see electronic supplementary material, figure S19) and northward spread (see electronic supplementary material, figure S20), the lack of new detections and/or introductions of new species in most other at-risk regions in the USA and Mediterranean Basin, and the high probabilities of repeatedly detecting many of the tephritid species in California while at the same time not detecting them in other at-risk areas. These findings do not rule out the possibility of multiple introductions into the state for tephritids such as the medfly [33,36–38]. However, the multiple detections of several species in nearly the same location anywhere from 10 to 30 years after they were first detected, without any captures during interim years, suggests that, as for many other invasive species [4,39,40], tephritids can be present in low numbers for decades [3,8,41–44]. Indeed, one of the important features of lags in invasion biology (i.e. the delayed onset or slow rate of an invasion event) that probably also applies to the tephritid invasion of California is that invasions are often not recognized until they are over [45,46].

Our findings that multiple species of tropical tephritids (including the Mediterranean, Mexican and oriental fruit flies, and possibly the peach, guava and melon fruit flies) have self-sustaining [41] and thus established populations in California have profound economic implications. For example, a 1995 study estimated that medfly establishment alone would result in $493 million to $875 million in annual direct costs, and the imposition of an embargo would cause an additional loss of $564 million. The state economy could lose $1.2 billion in gross revenue and more than 14 000 jobs [47].

However, two aspects of the invasions are advantageous for planners, programme directors and policy makers. The first is that local population sizes for all species are extremely small, and therefore likely to continue to be subdetectable. Therefore, based on phytosanitary standards of the International Plant Protection Convention [48,49], most regions of the state should continue to be classified as risk-free by trading partners. The second aspect of the invasions that can be exploited for the longer term involves the invasion lags, which imply that there can be relatively long windows of opportunity for developing new protocols and programmes. Commodity certification protocols can be developed for the creation of fly-free and low-prevalence zones [48], as can long-term research programmes on tephritid biology and management.

## 5. Implications for invasion science and policy

### (a) Early detection: a misleading misnomer

Because the likelihood of slowing the spread of or eradicating an alien pest depends heavily upon its residency time [50–52], a basic invasion biology canon is that early detection is critical for rapid response (but see [52]). Our results reveal that, because the sources of repeat detections are captures from established populations rather from reintroduced ones, in most cases ‘early detection’ is a misnomer when applied to tephritid detections at all scales. Because this expression is often inaccurate, it is also misleading inasmuch as it implies that a policy primarily directed at preventing new introductions will solve the problem of recurrent detections or infestations.

### (b) Rare-event detection problem

As is true for many alien insect populations [53], the majority of tephritid population growth and spread in the state is subdetectable because of the small size and cryptic habits of all life stages, the slow pace of naturalization processes, and suppression of populations by intervention programmes. In cancer diagnostics, this is referred to as the ‘rare-event detection problem*’* [54]; in the context of fruit fly detections, the parallel concept is the difficulty in discovering exceedingly rare, scattered, ultra-small populations of tephritids that are mostly in pre-adult stages, hidden among millions of properties and tens of millions of micro-niches. The scores of examples of repeat tephritid finds within a small region of California, separated by decades, suggest that the efficiency of detecting small populations of fruit flies is grossly overestimated [55], and that the actual chances of discovering populations that are so tiny and scattered is vanishingly small.

### (c) Cryptic invasion

Our findings are consistent with two interrelated invasion biology principles that underlie the ability of tephritid populations to establish and maintain residency at ultra-low, cryptic and insidious population levels. The first involves what Simberloff [4] refers to as the ‘mysterious lag phase’ in which new populations experience delayed growth [45]. It is unlikely that the magnitude of the lag period in tephritids would be similar to the 150+ years reported for some introduced plant species [39,56]. However, it is likely that tropical species of tephritids that are introduced to different climatic regions experience major population lags much like the multi-decade lags observed in the melon fly (*B. cucurbitae*) in Africa [57] and the cherry fruit fly (*Rhagoletis cingulata*) in Europe [43]. A second closely related principle is naturalization—genetic adaptation to local conditions [3]. Recent studies suggest that many species' invasion success may depend more heavily on their ability to respond to natural selection than on broad physiological tolerance or plasticity, and could also result from the need for multiple invasions to facilitate a sufficient evolutionary response [58].

### (d) Natural rather than human-enhanced spread

Although it is widely believed that human movement of infested plant material plays a major role in spreading introduced pests [6,11], capture patterns for the Mexican fruit fly suggest that this is not the case for this species and, by extension, may not be the case for many of the other invasive tephritids. For example, in 2011, 43 million vehicles and 17 million pedestrians crossed the six ports of entry from Mexico (where *A. ludens* is endemic) to California [59,60]. Assuming that the direction of movement for roughly half of these vehicles and people was from Mexico to California, and if humans entering and dispersing around the state were responsible for the Mexico-to-California as well as the within-state movement of the Mexican fruit fly, then this species should have been detected more or less randomly throughout the state. But the vast majority of all *A. ludens* detections for nearly 60 years have been in the same areas in which this species continually reoccurs. At the same time, there have been virtually no discoveries of *A. ludens* in the regions of the state with extraordinarily high movement of Latino populations (including tens of thousands of migrant workers), such as the main agricultural areas in the Central, Salinas and Imperial Valleys (see detailed local distributions in the electronic supplementary material, figure S1).

### (e) Principles of invasion biology and population theory

We know of no historical precedent in the invasion biology literature similar to the tephritid situation in California, where not only are several insect species within a single family (Tephritidae) invading a region at the same time, but the group also contains species within multiple genera. The California tephritid invasion thus provides unique opportunities to compare the invasive properties of species across different genera with similar life histories [61], to explore reasons why 17 tephritid species have been detected in California but few to none in many other fruit-fly-friendly regions of the USA and the world, and to develop new population theory for ultra-low, cryptic populations.

### (f) Conflation of criteria for eradication declaration

CDFA and USDA declared 100% success for each of the several hundred eradication programmes that were launched against fruit flies in California (see electronic supplementary material, table S1). These declarations were accurate according to legal criteria specified by the USDA [62] and the International Phytosanitary Commission [63]; that is, a region is declared (and thus certified) fruit-fly-free when no flies have been detected for a time period corresponding to three generations. Although these legal criteria are required for regulatory compliance to enable growers to ship their produce, our results reveal that the more stringent ecological requirements for eradication declaration were not met in the majority of cases. This underscores the continuing problem in the insect eradication literature of loosely and inaccurately applying a term (eradication) that has a clear definition. Those interested in insect eradication can learn much from the epidemiological literature on eradication programmes (e.g. malaria) regarding (i) frameworks for evaluating systematically the potential for eradication [64], (ii) clear definitions of concepts and terms [65], and (iii) perspectives on the preconditions, difficulties and challenges of successfully eradicating insects [66].

### (g) Population establishment: categories of likelihood

Although some authors have characterized population establishment as self-sustaining populations [35,67], none has attempted to specify criteria. The likely reason is that, because of the uncertainty resulting from a combination of demographic stochasticity and detection constraints, it is virtually impossible to define a precise point at which a small population becomes self-sustaining. In the light of this problem, we propose that early-stage invasions can be categorized using methods similar to those we used for Californian tephritids (see electronic supplementary material, table S7). The establishment category for each species is necessarily subjective and can be based on a combination of detection metrics, including capture span (i.e. period between first and last year captured), total number of years captured, inter-year frequency of detections (e.g. annually; bi-annually), total numbers of individuals detected, within-state distribution and spatial patterns of apparent spread.

## 6. Conclusions

Our results highlight the enormous historical challenges in addressing the continuing problem of invasive tephritids in California.

One the one hand, the challenge of population suppression appears to have been met and a level of control achieved repeatedly for most tephritids (excepting olive fly) through various CDFA- and the USDA-supported intervention programmes. That is, the current responses to the presence of fruit flies have been extraordinarily successful in reducing invasive fruit fly populations to levels that satisfy legal and regulatory requirements for keeping commodity trade routes open.

On the other hand, the greater challenge of invasive tephritids in California, and the one we show has not been achieved for most tephritid species in the state, is true biological (as distinct from legal) eradication—the complete elimination of the last vestige of a population.

The long-term consequences of making short-term policy decisions based solely on the legal definition of eradication, while ignoring the biological reality (i.e. fruit fly establishment and spread), are potentially quite serious. Indeed, the call more than 20 years ago for decisive leadership to deal with medfly establishment in California because ‘the pest cannot be wished away or legislated out of existence’ [22, p. 516] now also applies to a number of the medfly's closest relatives.
